# Supplementary material for: Evidence supported by Mendelian randomization: impact on inflammatory factors in knee osteoarthritis
Source: Front Med (Lausanne). 2024 May 28;11:1382836. doi: 10.3389/fmed.2024.1382836 (PMC11165061; doi:10.3389/fmed.2024.1382836)
Supplement: Supplementary file 1 [file Data_Sheet_1.ZIP › data/GCST90274845_5e6/845.forest.pdf]

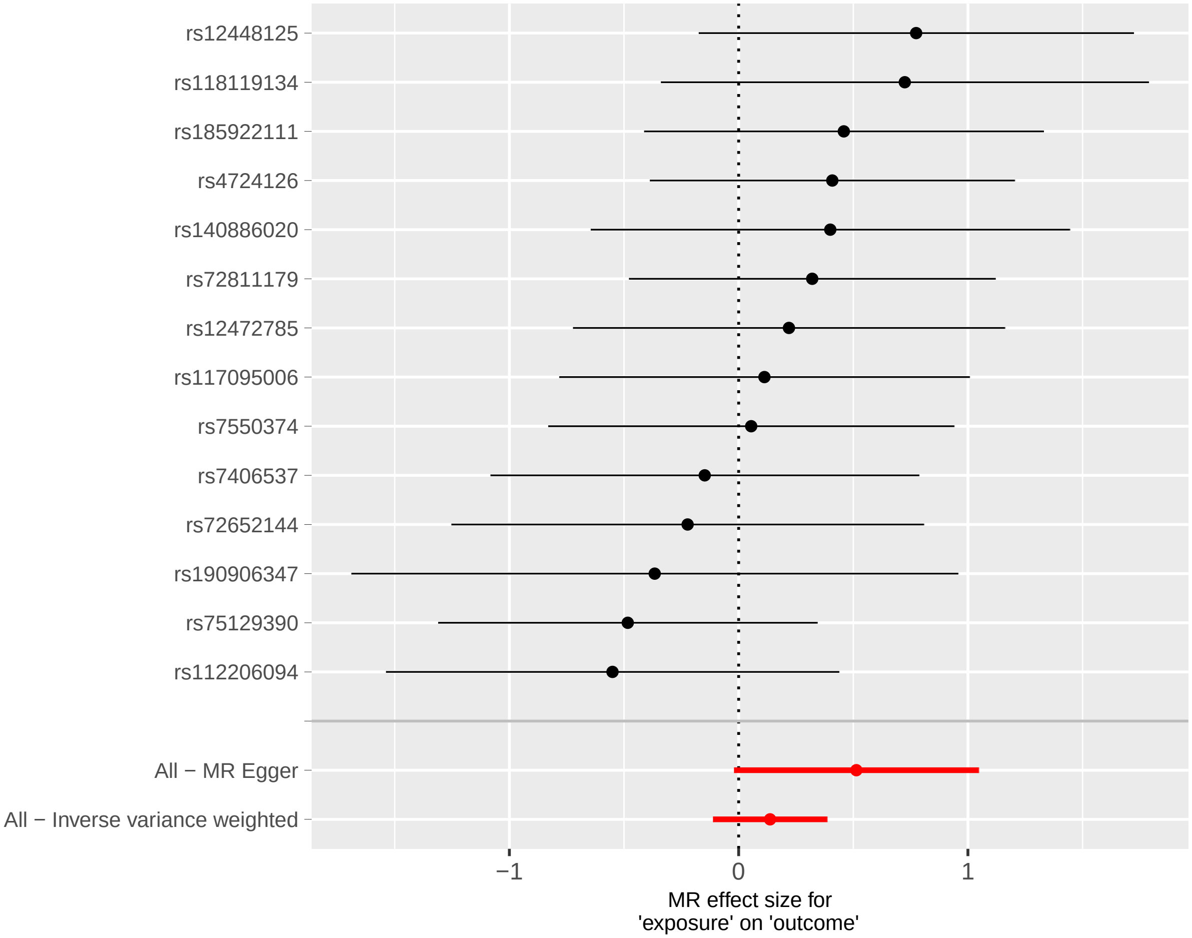

rs12448125
rs118119134
rs185922111
rs4724126
rs140886020
rs72811179
rs12472785
rs117095006
rs7550374
rs7406537
rs72652144
rs190906347
rs75129390
rs112206094
All − MR Egger
All − Inverse variance weighted
−1
0
1
MR effect size for
'exposure' on 'outcome'
